# Primary bipolar hemiarthroplasty as a treatment option for unstable intertrochanteric fractures

**DOI:** 10.20407/fmj.2019-022

**Published:** 2020-03-25

**Authors:** Kosuke Tajima, Masahiro Yoshida, Daiki Murakami, Tomoyuki Nishimura, Akihiko Hirakawa, Norimichi Uenishi, Mitsunaga Iwata

**Affiliations:** 1 Department of Emergency Medicine, Fujita Health University Hospital, Toyoake, Aichi, Japan; 2 Department of Surgery, Tokyo Hokubu Hospital, Tokyo, Japan; 3 Department of Disaster Medicine and Traumatology, Fujita Health University, School of Medicine, Toyoake, Aichi, Japan; 4 Department of Emergency and General Internal Medicine, Fujita Health University Hospital, Toyoake, Aichi, Japan; 5 Department of Emergency and General Internal Medicine, Fujita Health University, School of Medicine, Toyoake, Aichi, Japan

**Keywords:** Primary bipolar hemiarthroplasty, Unstable intertrochanteric fractures, Harris Hip Score

## Abstract

**Objectives:**

Management of unstable intertrochanteric fractures is challenging, especially in patients with osteoporosis. Comminuted unstable intertrochanteric fractures require postoperative immobilization. Several recent reports have recommended hemiarthroplasty for treatment of unstable intertrochanteric fractures to avoid various immobilization-associated complications. The purpose of this study was to evaluate the functional and clinical outcomes of bipolar hemiarthroplasty for unstable intertrochanteric fractures in older persons.

**Methods:**

Sixty patients aged over 75 years underwent hemiarthroplasty to treat unstable intertrochanteric fractures and were followed up over 12 months. All surgeries were performed by the same surgical team using the standard posterolateral approach. Wires, cables, and plates were used as required. Use of cemented protheses was considered when the lesser trochanter had been displaced. All patients were allowed full weight-bearing as tolerated. Clinical evaluation was based on Harris Hip Scores.

**Results:**

The cohort comprised 16 men and 44 women (aged 75–96 years). According to the Jensen classification, 24 fractures were type III, 14 type IV, and 22 type V. Cement was used in 24 patients. At 12 months follow-up, Harris Hip Scores were excellent in 18%, good in 42%, fair in 25%, and poor in 15%. No radiological abnormalities were detected.

**Conclusions:**

Primary bipolar hemiarthroplasty for treating unstable intertrochanteric fractures eliminates the need for prolonged immobilization and permits early ambulation. As reported by others, hip hemiarthroplasty is an effective treatment choice for unstable intertrochanteric femoral fracture in older patients.

## Introduction

Intertrochanteric fractures comprise approximately 45%–50% of all hip fractures in older persons^1^ and 50%–60% of them are classified as unstable.^2^ Unstable intertrochanteric fractures are of major cause of concern in older patients because of the associated high morbidity and mortality.^3^

Intramedullary nailing is the treatment of choice for stable hip fractures. Intramedullary nailing techniques require only a small incision and protect patients’ bone structure. Intramedullary nailing reduces surgical complications, blood loss, and infection.^4^ Thus, the minimally invasive procedure of intramedullary nailing is considered the most appropriate for geriatric patients.

Management of unstable intertrochanteric fractures is challenging in older patients because of their poor bone quality and high risk of morbidity and mortality.^5^ Osteoporosis and instability are two of the most important factors leading to unsatisfactory treatment outcomes.^6,7^

Selection of the implant type is extremely important because it affects these patients’ survival and functional outcomes. Intramedullary nailing is also the most commonly performed procedure for unstable intertrochanteric fractures; however, a review of published reports indicates there is a lack of consensus regarding this choice.^4^

**Figure 1:**
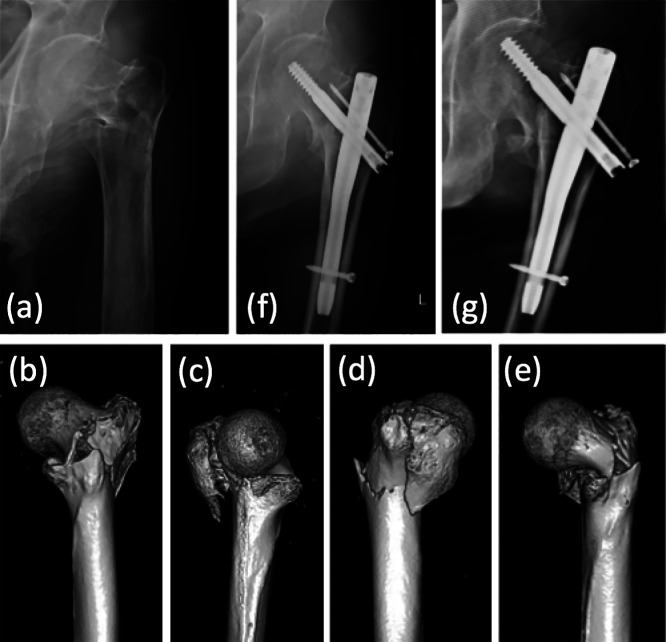
This 84-year-old man fell to the floor and sustained a type III intertrochanteric fracture (Jensen classification) of the left hip. Preoperative anteroposterior radiograph (a) and 3D-CT images (b–e). Surgery with proximal femoral nailing was performed (f); however, dislocation of the fracture occurred only 1 month later (g). (This case is not included in the study cohort.)

Early postoperative resumption of full weight-bearing is difficult after intramedullary nailing because of the combination of an unstable fracture pattern, osteoporosis, and the tendency of geriatric patients to have restricted mobility for various reasons.^8^ Internal fixation of unstable fractures may be accompanied by problems such as collapse, loss of fixation, and cut-out that lead to impaired function (Figure 1).^9^ Many surgeons have therefore recently suggested hemiarthroplasty to allow early full loading and prevent collapse in the fracture area.^8,10–16^

The purpose of this study was to evaluate the functional and clinical outcomes of bipolar hemiarthroplasty as the primary treatment for unstable intertrochanteric fracture in older patients.

## Material and Methods

This retrospective study was conducted in accordance with the guidelines of the ethics committees of our institutes.

Of the 760 proximal femoral fractures in ambulatory patients treated surgically between 2013 and 2018 in our institutes, 66 unstable intertrochanteric fractures were treated by hemiarthroplasty and 60 of them were followed up over 12 months.

All study patients was aged over 75 years. Anteroposterior X-ray films of the pelvis, lateral X-ray films of the hip joint, and CT scan of the pelvis with multi planner reconstruction and 3D views were taken in all cases. The patients underwent elective surgery after management of avoidable anesthetic risks. The surgeries were performed under spinal or general anesthesia.

The fractures were classified according to the Jensen–Michaelsen classification.^17^

**Figure 2:**
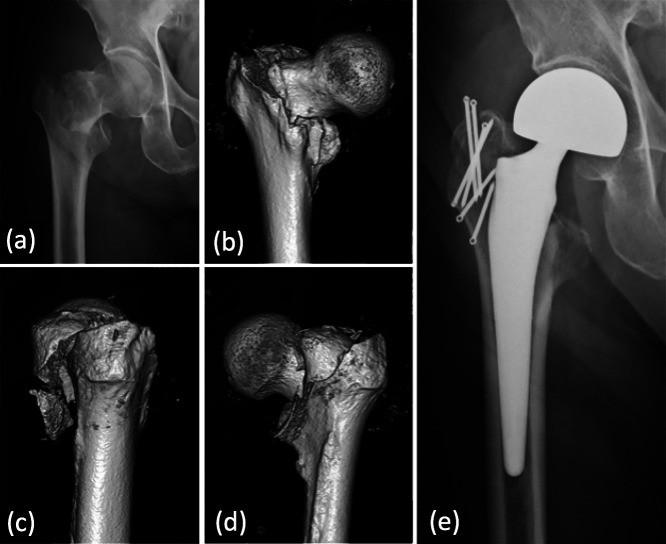
This 93-year-old man fell to the floor while walking with a walker and sustained a type III intertrochanteric fracture (Jensen classification) of the right hip. Preoperative anteroposterior radiograph (a) and 3D-CT images (b–d). Uncemented hemiarthroplasty with ring pins with polyethylene cables were used to reconstruct the greater trochanter (e). Full weight-bearing was permitted from the day after surgery. The patient’s outcome 12 months after the surgery was graded as fair.

**Figure 3:**
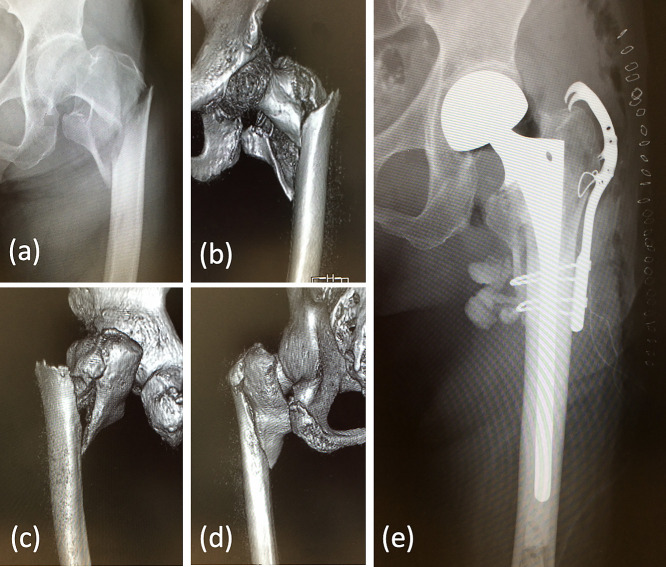
This 86-year-old woman fell while riding a bicycle and sustained a type V intertrochanteric fracture (Jensen classification) of the left hip. Preoperative anteroposterior radiograph (a) and 3D-CT images (b–d). Cemented hemiarthroplasty with plate and wire loops were used to reconstruct the medial and lateral buttresses (e). Full weight-bearing was permitted from the day after surgery. The patient’s outcome 12 months after the surgery was graded as excellent.

All surgeries were performed by the same surgical team using the standard posterolateral approach. After assessing the fracture, a T-shaped incision was made in the joint capsule and the femoral head was removed. In cases of posteromedial comminution, reconstruction was achieved using stainless steel surgical wires, Kirschner wires, or polyethylene cables, after which a cemented or uncemented bipolar prosthesis was inserted. In cases of comminution of the greater trochanter, stainless steel surgical wires, Kirschner wires, polyethylene cables, ring-pins or plates were used for reconstruction (Figures 2, 3). Use of cemented protheses was considered when the lesser trochanter had been displaced (Figure 3).

Antibiotics were administered only on the day of surgery. Postoperatively, adequate analgesics were given. The affected limb was kept in 30° of abduction with the help of a pillow between the thighs for 2 weeks.

All patients were referred to the rehabilitation department for prevention of disuse syndrome on admission, and allowed full weight-bearing as tolerated from the day after surgery. Sutures were removed 2 weeks after surgery. Patients were followed up for 12 months or more postoperatively and assessed both clinically and radiologically. Clinical evaluation was based on Harris Hip Scores,^18^ scores of <70 being classified as poor, 70–79 as fair, 80–89 as good, and 90–100 as excellent.

## Results

Six of the 66 patients were excluded from the study because they died of non-surgery related causes. Thus, 60 patients were evaluated in this study.

The 60 patients comprised 16 men (27%) and 44 women (73%), and were aged from 75–96 years (average 86). Only 43 patients (72%) could walk without any support prior to the injury.

According to the Jensen–Michaelsen classification, 24 fractures were type III, 14 type IV, and 22 type V. Cement was used in 24 patients.

Surgery was performed under general anesthesia in 36 cases and spinal anesthesia in 24 cases. The average duration of surgery was 66.1±25.9 minutes (range 40–145). Additional wiring was used in 52 cases and additional plates in seven. The average volume of intraoperative hemorrhage was 207±145 mL (range 12–459) and 47 patients (78%) required blood transfusion, the total average amount transfused on the day of surgery and the following day being 2.1±1.8 units (range 0–6). The average duration of hospital stay was 24.5±15.0 days (range 6–59). Five patients were discharged home and 55 were transferred to nearby hospitals for rehabilitation. No dislocations occurred; however, two patients required additional surgeries because of periprosthetic fracture caused by falling out of bed. No surgical site or deep infections, pulmonary embolism (PE), deep vein thrombosis (DVT), or pneumonia occurred in this series; however, six patients had urinary tract infections (UTI).

At 12 months follow up, the outcomes of 11 patients (18%) were graded as excellent, of 25 (42%) as good, of 15 (25%) as fair, and of 9 (15%) as poor according to Harris Hip Scores (Table 1). No radiological abnormalities were detected.

## Discussion

Management of unstable intertrochanteric fractures remains controversial, especially in patients with osteoporosis.^19^ Stable intertrochanteric fractures can easily be treated by osteosynthesis with predictable good results.^2,20,21^ Internal fixation achieves good results in stable fractures with good bone stock; however, all types of fixation devices have failed to achieve the same success rate in comminuted unstable and osteoporotic intertrochanteric fractures.^22^ The biological and biomechanical changes associated with osteoporosis make the management of these fractures more difficult. Cortical bone becomes thin and cancellous bone has reduced bone mineral density with changes in the trabecular pattern. Thus, the management of unstable intertrochanteric fractures is challenging because of poor bone quality and osteoporosis, as well as other age-associated comorbidities.^7,21^

Hip fractures are associated with notable morbidity and mortality in older patients. Rigid fixation and early rehabilitation can drastically reduce the mortality associated with intertrochanteric fractures.

Internal fixation in patients with age-related osteoporosis often has a high failure rate because of the poor bone quality,^23^ hindering early mobilization. The incidence of general complications such as PE, DVT, and pneumonia ranges from 22%–50% when mobilization is delayed after internal fixation.^24,25^

Restoration of mobility depends on the quality of bone and the type of implant used. In cases of comminuted fractures, fixation of the fragments is difficult and the posteromedial void in this region makes the fracture very unstable. The incidence of failure with unstable intertrochanteric fractures is as high as 50%,^2^ and the cut-out rate can be high as 4%–16.5% for hip screws.^23,26,27^ Thus, rigid internal fixation and early mobilization are the keys to successful treatment.

To enable early postoperative weight-bearing and rapid rehabilitation, several authors have recommended hemiarthroplasty for treatment of unstable intertrochanteric fractures.^8,10–16^

Tronzo^28^ claimed to be the first to use long, straight-stemmed prostheses for the primary treatment of intertrochanteric fractures. Subsequent studies also achieved good results with hemiarthroplasty.

In a comparative study of hemiarthroplasty versus internal fixation, Kayali et al.^19^ concluded that the clinical results of these procedures are similar. Grimsrud et al.^29^ also reported good outcomes.

Esen et al.^30^ compared proximal femoral nail-antirotation (PFNA) and cemented arthroplasty. They reported that duration of surgery, units of blood transfusion, and duration of hospital stay were less for PFNA; however, there was no significant difference in postoperative complications, Harris hip scores, or one-year mortality.^30^ They concluded that these techniques for performing bipolar hemiarthroplasty have equivalent outcomes in patients in an acceptable general condition.

Mansukhani et al. randomized 60 intertrochanteric fractures into three groups, and compared the clinical results of cemented hemiarthroplasty, dynamic hip screws, and proximal femoral nails, and found no significant disadvantage for arthroplasty.^27^

Patients who have undergone hemiarthroplasty are permitted immediate mobilization; thus, rehabilitation is quick and there are markedly fewer complications related to prolonged immobilization, such as decubitus ulcers, respiratory infection, and atelectasis.^31^ Treatment of unstable intertrochanteric fracture in older patients with bipolar hemiarthroplasty results in fewer complications of prolonged immobilization and easy rehabilitation along with a rapid return of functional level. Rodop^32^ also reported a successful result.

Treating unstable intertrochanteric fractures in older patients with compromised general health and comminuted fractures in osteoporotic bone stock by primary hemiarthroplasty essentially bypasses the phase of fracture healing by immediately providing a stable, mobile, relatively pain-free joint.^8^ This eliminates the need for prolonged immobilization and permits early ambulation. Davison et al. have also reported that cemented hemiarthroplasty provides the advantage of load bearing ambulation in the early post-operative period.^9^

There are reports that delayed surgery leads to muscle mass attenuation by 0.5%–0.6% per day and muscle strength attenuation by 2%–4% per day.^33,34^ Furthermore, Yamaguchi et al. reported that delaying starting walking rehabilitation results in an additional 2.8 days per days delayed to achieve the ability to walk.^35^ We have not used conventional surgical techniques that require a long period of non-weight-bearing to treat unstable intertrochanteric femoral fractures and therefore could not perform a comparative study. However, the functional results our patients achieved are comparable to those reported by others, as shown in Table 2. These studies had similar functional results and all concluded that hemiarthroplasty is a beneficial treatment for unstable intertrochanteric femoral fractures.

To the best of our knowledge, no Japanese studies have yet assessed the outcomes of this surgical procedure. In summary, hip hemiarthroplasty is an effective salvage procedure and is the treatment of choice for unstable intertrochanteric femoral fractures in older patients.

## Figures and Tables

**Table1:** Result of bipolar hemiarthroplasty for unstable intertrochanteric fractures

Cases	60 (16 males, 44 females)
Age (years)	75–96 (average 86)
ADL before injury	walk without support or cane for long walk: 43 with cane, crutch or walker: 14 wheel chair or cannot walk: 3
Fracture type (Jensen-Michaelsen classification^17^)	III: 24 IV: 14 V: 22
Type of anesthesia	General: 36 Spinal: 24
Surgical time (minutes)	40–145 (average 66±26)
Intraoperative hemorrhage (mL)	12–459 (average 207±145)
Blood transfusion (on the day and the next day of surgery)	47 cases 0–6 units (average 2.1±1.8)
Surgical site infection	None
Harris Hip Score^18^ (at 12 months)	Excellent: 11 (18%) Good: 25 (42%) Fair: 15 (25%) Poor: 9 (15%)
ADL after surgery (at final follow up)	walk without support or cane for long walk: 30 with cane, crutch or walker: 20 wheel chair or cannot walk: 10

**Table2:** Comparison to other studies of Harris Hip Score at 12 months

	Mansukhani^27^	Rawate^8^	Yadav^15^	Haentjens^11^	Sancheti^20^	Allam^16^	Rodop^32^	Our study
n=	10	30	27	30	33	27	37	60
Excellent	30 (%)	26.7	29.6	23.3	24.2	37.0	45.9	18.3
Good	40	33.3	66.7	36.7	48.5	44.4	37.8	41.7
Fair	20	33.3	3.7	23.3	18.2	18.5	8.1	25.0
Poor	10	6.7	0	16.7	9.1	0	8.1	15.0
